# Research ethics in context: understanding the vulnerabilities, agency and resourcefulness of research participants living along the Thai–Myanmar border

**DOI:** 10.1093/inthealth/ihaa052

**Published:** 2020-11-09

**Authors:** Napat Khirikoekkong, Nattapat Jatupornpimol, Suphak Nosten, Supa-at Asarath, Borimas Hanboonkunupakarn, Rose McGready, Francois Nosten, Jennifer Roest, Michael Parker, Maureen Kelley, Phaik Yeong Cheah

**Affiliations:** Shoklo Malaria Research Unit, Faculty of Tropical Medicine, Mahidol University, Mae Sot, Thailand; Mahidol Oxford Tropical Medicine Research Unit, Faculty of Tropical Medicine, Mahidol University, Bangkok, Thailand; Mahidol Oxford Tropical Medicine Research Unit, Faculty of Tropical Medicine, Mahidol University, Bangkok, Thailand; Shoklo Malaria Research Unit, Faculty of Tropical Medicine, Mahidol University, Mae Sot, Thailand; Mahidol Oxford Tropical Medicine Research Unit, Faculty of Tropical Medicine, Mahidol University, Bangkok, Thailand; Department of Clinical Tropical Medicine, Faculty of Tropical Medicine, Mahidol University, Bangkok, Thailand; Shoklo Malaria Research Unit, Faculty of Tropical Medicine, Mahidol University, Mae Sot, Thailand; Centre for Tropical Medicine & Global Health, Nuffield Department of Medicine, University of Oxford, Oxford, UK; Shoklo Malaria Research Unit, Faculty of Tropical Medicine, Mahidol University, Mae Sot, Thailand; Centre for Tropical Medicine & Global Health, Nuffield Department of Medicine, University of Oxford, Oxford, UK; Wellcome Centre for Ethics & Humanities, Nuffield Department of Population Health, University of Oxford, Oxford, UK; The Ethox Centre, Nuffield Department of Population Health, University of Oxford, Oxford, UK; Wellcome Centre for Ethics & Humanities, Nuffield Department of Population Health, University of Oxford, Oxford, UK; The Ethox Centre, Nuffield Department of Population Health, University of Oxford, Oxford, UK; Wellcome Centre for Ethics & Humanities, Nuffield Department of Population Health, University of Oxford, Oxford, UK; The Ethox Centre, Nuffield Department of Population Health, University of Oxford, Oxford, UK; Mahidol Oxford Tropical Medicine Research Unit, Faculty of Tropical Medicine, Mahidol University, Bangkok, Thailand; Centre for Tropical Medicine & Global Health, Nuffield Department of Medicine, University of Oxford, Oxford, UK; The Ethox Centre, Nuffield Department of Population Health, University of Oxford, Oxford, UK

**Keywords:** agency, consent, migrants, pregnant women, research ethics, vulnerability

## Abstract

**Background:**

Research ethics guidelines set a high bar for conducting research with vulnerable populations, often resulting in their exclusion from beneficial research. Our study aims to better characterise participants’ vulnerabilities, agency, resourcefulness and sources of support.

**Methods:**

We undertook qualitative research around two clinical studies involving migrant women living along the Thai–Myanmar border. We conducted 32 in-depth interviews and 10 focus group discussions with research participants, families, researchers and key informants.

**Results:**

We found that being ‘undocumented’ is at the core of many structural vulnerabilities, reflecting political, economic, social and health needs. Although migrant women lead challenging lives, they have a support network that includes family, employers, community leaders, non-governmental organisations and research networks. Migrant women choose to participate in research to access quality healthcare, gain knowledge and obtain extra money. However, research has the potential to exacerbate existing vulnerabilities, such as the burdens of cross-border travel, foregoing work and being more visible as migrants.

**Conclusions:**

Our study confirms that research is important to provide evidence-based care and was viewed by participants as offering many benefits, but it also has hidden burdens. Migrant women exercised agency and resourcefulness when navigating challenges in their lives and research participation.

## Introduction

To be vulnerable is to be more susceptible to risks and less able to protect one's own interests.^1^^,^^2^ Being in a vulnerable situation shapes obligations for others to help or take special care. In research, a participant's vulnerability creates special obligations to protect participants’ safety and interests, such as the requirement for parental or surrogate consent for enrolling children and inclusion of a prisoner or prisoner representative on ethics review boards that assess research involving prisoners.^3^^–^^5^ Historically, international guidelines have conceptualised vulnerability as an attribute of groups or populations, e.g. neonates, children, prisoners, persons living in poverty and others at risk of being exploited.^5^^–^^7^ However, by classifying a population as vulnerable we can err in two ways. First, by labelling ‘pregnant women’ or ‘migrants’ or ‘children’ as vulnerable,^8^^,^^9^ we may be too restrictive and unfairly exclude some who are capable of consenting or assenting with support^10^ and could potentially directly benefit from the research or benefit from knowledge gained by studying that group's particular health needs. For example, the underinclusion of pregnant women and children who have malaria has resulted in decades of underdosing of malaria drugs for these groups.^9^ Second, we might not be protective enough, overlooking hidden vulnerabilities of individuals who do not belong to designated vulnerable groups but, for example, may lack social support.^11^ For these reasons we have seen calls for more emphasis on context and greater specification of the ways in which particular research participants might be vulnerable to inform more responsive research protections.^12^^,^^13^

This change is welcome, particularly in low-resource settings where research is needed to address critical health needs but where potential research participants experience multiple sources of vulnerability. Structural sources of vulnerability such as poverty, literacy skills, stigma, lack of political power or status and gender inequality contribute to participants’ greater susceptibility to coercion or inability to say no to research when there are benefits from research, such as access to clinical care or treatment. However, we need empirical work to understand how these factors manifest in the research encounter to better inform this proposed shift in research ethics from a focus on ‘vulnerable populations’ to special scrutiny of the circumstances in which research occurs.^11^^,^^14^ Specifically, there are important gaps in our understanding of what happens during research with those who may be vulnerable, what sources of support are available and how we might better engage the agency or resourcefulness of people who are vulnerable in some ways but not others.

To address this evidence gap, we conducted an international empirical ethics study, ‘Resilience, Empowerment and Advocacy in Women's and Children's Health Research’ (REACH) in Kenya, South Africa and Thailand to better understand both the potential benefits and ethical challenges of research with participants with the types of complex vulnerabilities that characterise many participants in global health research. This article reports the findings of the case study that focused on migrants and pregnant women living in the politically volatile Thai–Myanmar border region. We investigated how migrant women's complex vulnerabilities of daily living intersected with participation in research. We explored the harms and risks migrant pregnant women are vulnerable to, from their own perspectives, how they exercise agency related to daily living and research participation and where they find sources of support in the context of health research.

### Setting

Our study was undertaken in the Tak Province in northwestern Thailand, which shares a long and porous border with Karen State, Myanmar, demarcated by the Moei River. The Tak Province and Karen State on either side of the border are mountainous regions with dirt roads, heavily forested areas and rice fields.

Since the 1980s, political and militarised ethnic conflicts within Myanmar have forced hundreds of thousands of people from Myanmar, especially ethnic minorities, to take shelter in Thailand. In addition to conflict, economic stagnation in Myanmar has also driven millions of migrant workers to Thailand in search of work, healthcare and other essential needs. Migrant status can be categorised as ‘stable’, ‘cross-border’ and ‘unstable’ (Table 1). Persons who have been granted refugee status are not considered migrants.

**Table 1.** Profile and characteristics of three different types of migrants on the Thai–Myanmar border. ‘Unstable’ migrants are the most vulnerable

Group	Documented	Undocumented	
Type	Stable	Cross-border	Unstable
Shared characteristics	From Myanmar		
	Mixture of Burmese, Karen and other ethnicities		
	Language: Karen, Myanmar and some can speak some Thai		
	Low literacy		
	Earning below-average wages (120–150 baht/day [US$4–5]		
	Mixture of nuclear and extended family units		
Type of employment	Agriculture		
	Casual labour		
	Construction		
	Domestic work		
Living arrangements	Living on the Thai side of the border	Living on the Myanmar side of the border	Living on the Thai side of the border
	Employer provides accommodation and safety	Picked up for casual daily labour	Self-arranged, variable accommodation
			High mobility
Access to healthcare	Thailand primary care units (PCUs)/subdistrict health promotion hospitals (SHPHs) but must pay if they have no documentsThai local hospitals	No healthcare entitlement in ThailandVisit the SMRU for antenatal care/other healthcare services	No healthcare entitlement in ThailandNot much knowledge about self-care
	Mae Tao Clinic (NGO clinic)	Community health groups	Decreased access to healthcare facilities due to travel and costs barriers
		Mae Tao Clinic	

Our study site was the Shoklo Malaria Research Unit (SMRU), a field research site of the Bangkok-based Mahidol–Oxford Tropical Medicine Research Unit (MORU). The SMRU has its offices in Mae Sot, Tak Province, and clinics located on both sides of the border. We chose this site not only for the opportunity to understand the complex vulnerabilities of migrant women in research but to understand how research institutions might be designed to respond to such needs through research, ancillary care and evidence-based humanitarian services.^15^^,^^16^ The SMRU has provided free humanitarian healthcare, including for mothers and children, for more than 30 years. Its clinics and laboratories are staffed by a combination of foreign doctors and researchers and local Karen and Burmese healthcare workers. The majority of patients served by the SMRU are migrant workers and refugees from Myanmar. Thai nationals do not tend to access the SMRU clinics since they have universal health coverage and can access Thai government hospitals.

It has been estimated that there are one million undocumented migrants in Thailand not covered by any government health insurance scheme. To address this problem, the Migrant Fund (M-Fund), a non-profit insurance scheme, was co-developed in 2017 by the SMRU with support from the Thai Ministry of Health to provide health coverage for undocumented migrants. M-Fund members contribute a low monthly premium of 100 Thai baht (US$2.8) per person, which covers access to broad quality healthcare services in a network of partner hospitals. In addition, this fund helps provide ancillary care that is beyond the capacity of the SMRU clinics, including health issues requiring referral to hospitals.

## Materials and methods

The research reported here embedded social scientists and bioethicists within a clinical research team at the SMRU,^17^ linked to two approved clinical studies, one on treatment of uncomplicated malaria during pregnancy (completed and submitted for publication) and another on hepatitis B prevention during pregnancy (ongoing). Table 2 describes key features of the linked studies.

**Table 2.** Key features of linked clinical studies in the REACH research ethics study

Study title acronym	DMA Study	TDF Study
Clinical trials identifier (on ClinicalTrials.gov)	NCT01054248	NCT02995005
Study objectives	To determine the efficacy and safety of dihydroartemisinin–piperaquine, artesunate–mefloquine and artemether–lumefrantrine (augmented dose) in the treatment of uncomplicated malaria in pregnant women	To estimate the time to complete hepatitis B virus (HBV) DNA suppression in 170 HBV DNA–positive women who started tenofovir in the late first or early second trimester and to estimate the proportion of women with HBV DNA at delivery
Study design	Randomised 1:1:1, open label	Single-arm, open-label, tenofovir treatment intervention study
Study period	October 2009–December 2018	May 2017–December 2021
Participants	Pregnant women in first, second and third trimester with acute uncomplicated malaria, ages 18–45 y, and their offspring	Pregnant women estimated gestation 12–<20 weeks, ages 16–45 y, and their offspring
Sample size	511 (actual)	170 (estimated)
Key study procedures	Follow-up from enrolment in pregnancy to infant age 4 y	Monthly visits from enrolment to infant age 2 months, then again at age 4 and 6 months
	Daily follow-up until malaria smear negative, then weekly to day 63, then every 1–2 weeks until deliveryInfant: monthly in year 1 then every 3 months thereafter. Small-volume finger prick samples (×10) for drug level analysis in first 42 days	Monthly venous blood draw for safety (mother kidney and liver test) and HBV viral load in mother and single venous blood draw for baby (at 2 months of age)
		
Risks	All drugs used in the study are recommended by international malaria treatment guidelines. The dose of artemether–lumefantrine is higher in the study than in non-pregnant women. There may be side effects related to the higher dose	Risk of liver flare due to disease, but this is increased when the drug is ceased post-partum. It is usually biochemical without symptoms, but can be severe and is treatable
Direct benefits	No additional direct benefit, as patients are treated for free with the same drugs (dihydroartemisinin–piperaquine, artesunate–mefloquine) in the same clinics	Tenofovir is not readily accessible outside of the study and for this population is relatively expensive—about 1300 baht (US$ 40) per month, not including liver tests, approximately 11 d of salary, which is substantial for a migrant family. Off-patent formulations may be cheaper if accessible in Thailand
		Knowing HBV status
Compensation	100 baht (US$3) per follow-up visit	50 baht (US$ 1.50) per follow-up visit
	Transportation costs	Transportation costs
Potential benefits to the population of pregnant women	Data will potentially inform improved targeted treatment of uncomplicated malaria in pregnant women	Exploring these kinetics is critical for maximizing the efficacy and efficiency of any antiviral interventions during pregnancy
Sponsor; funder	University of Oxford; Holleykin Pharmaceuticals (with core funding by the Wellcome Trust)	University of Oxford; Thrasher Research Fund (with core funding by the Wellcome Trust)

DMA, randomised trial of 3 artemisinin combination therapy for malaria in pregnancy; TDF, tenofovir in early pregnancy to prevent mother-to-child transmission of hepatitis B virus.

From December 2017 to March 2019 we conducted 32 in-depth interviews (IDIs) and 10 focus group discussions (FGDs) with four groups of participants (Table 3). All participants in our study were ≥18 y of age and provided written consent in their own language if literate or verbal consent in the presence of a literate impartial witness, if illiterate. The lead interviewers (NK, SN), who were independent of the linked clinical studies, are native Karen/Burmese speakers and have a deep understanding of the research setting and cultural context. Data collection continued until a point of saturation when it was determined new themes were unlikely to emerge.

**Table 3.** Description of REACH study participants by group with breakdown of participants in each group by gender and data collection methods

Participant group	IDIs	FGDs	
Group 1: Pregnant women participating or have participated in the linked studies and partners or supporting family members			
Linked study participants (DMA)	Female	7	4 groups (12 persons, all DMA participants)
Linked study participants (TDF)	Female	3	(4 participants joined both IDI and FGD)
Linked study participants’ key supporter (of DMA participants)	Male	2	
Group 2: Research physicians, study team and other researchers involved in the recruitment process, implementation and coordination of the linked studies			
Researchers/frontline healthcare workers	Female	6	4 groups (28 persons)
	Male	2	
Group 3: T-CAB and local ethics committee members. The T-CAB is a community advisory board established in 2009 that advises researchers on practical and ethical aspects of research and health programmes on the Thai–Myanmar border			
T-CAB and ethics committee	Female	1	2 groups (8 persons)
	Male	3	
Group 4: Persons who are knowledgeable about health on the Thai–Myanmar border, such as community leaders, government organizations and NGOs			
Key informants (village chiefs, elders, NGO workers)	Female	1	–
	Male	5	
Total		32 persons	10 groups

The IDIs and FGDs were conducted using a topic guide in English with translations into Myanmar and Karen. All interviews were audio recorded and translated into English. Any ambiguities in the original language were discussed across the team to ensure fidelity to the participants’ meanings in social and cultural contexts. The concept of ‘vulnerability’ did not have a direct Karen/Myanmar translation, so careful probing around ‘challenges’ was used. For example, the Karen words used to describe the meaning of ‘challenges’ and/or ‘vulnerability’ included barrier (*tar-ti-tar*), burden (*ta-wee-ta-yoh*) and a heavy load to carry (*ta-wee-kher*).

Analysis began as soon as the first interviews were transcribed and continued throughout the study in tandem with data collection. De-identified transcripts were imported to NVivo software (QSR International, Melbourne, VC, Australia) to organize and manage qualitative data.

In addition to IDIs and FGDs, we conducted a participatory visual workshop (PVM)^18^ with members of the Tak Province Community Ethics Advisory Board (T-CAB)^19^^–^^21^ and co-created drawings reflecting the themes that had emerged from prior interviews. We include drawings from that workshop here.

## Results

Our results describe the intersection of complex structural vulnerabilities of daily living for migrants and the benefits and hidden burdens of research in this border region. Even within a research programme with a long-standing commitment to prioritising provision of humanitarian care, these vulnerabilities gave rise to often distressing ethical dilemmas for researchers when balancing the benefits and burdens of research, determining the scope of duties of care and ensuring voluntary participation. Despite difficult life challenges, migrant women exercised agency and resourcefulness in daily life and research participation.

### Structural vulnerabilities and sources of support

In order to understand participants’ experience of vulnerability in clinical research, we sought to first understand participants’ challenges and sources of support in their daily lives. Consistent with established research on migrants in this region, we found that being legally ‘undocumented’ was at the core of many vulnerabilities. Being undocumented means there is a risk of being arrested, which could result in either paying a bribe to the authorities or worse, deportation to Myanmar.

**Figure 1.**
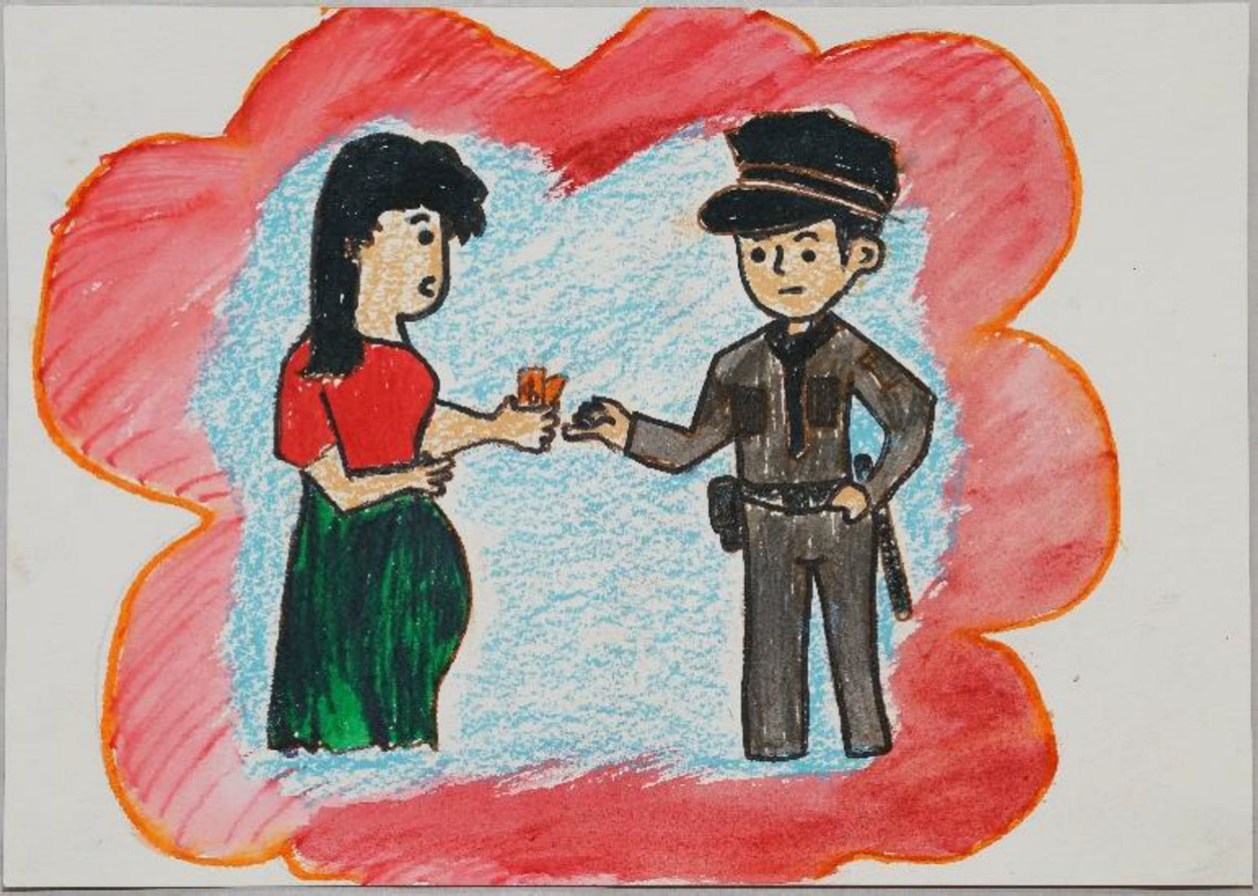
A drawing by the T-CAB members during a participatory visual workshop illustrating that many migrants have to spend extra money in the form of ‘fees’ or bribes to pass checkpoints.

To avoid getting arrested by local police, many migrants have to pay ‘fees’ or bribes to pass checkpoints and get employment, as depicted in this drawing by a T-CAB member (Figure 1) and the quote below.

*I: Who brought you there [Bangkok]?*

*0058: Backdoor path [laughter]…situation was not good…cost for a passport and all the other cards were so expensive. The police officer often came. (IDI, TDF participant)*

Lack of education was seen as a root cause of ongoing difficulties finding stable work and providing for one's family.

*0019: My hope is for the Karen people to have stable and free education opportunities so they can learn…even when we completed grade 10, we still cannot find stable job with good salary. We continue to face hardships with our employment situation. This is due to lack of education which affects the stability of our income and how inadequate we can provide for our families. (FGD, T-CAB member)*

Women at the border are also particularly vulnerable to domestic violence during seasonal lapses in work when husbands are unemployed and at home. Many men struggle with gambling, alcohol and substance abuse, which not only perpetuates poverty but leads to health problems, family disputes and domestic violence, as the woman below shared:

*0014: Fighting between me and my husband…it is very unfortunate for me to have married him; he is using amphetamines, and he is alcoholic. He disappeared between 4–5 days for work…It makes me feel sad. (FGD, DMA participant)*

Legal status also has important implications for whether patients are eligible to receive free or affordable care. Participants explained that general healthcare entitlements differed significantly between refugees and migrants. Within the migrant group there are also differences between stable, cross-border and unstable migrants. Only documented migrants have access to Thai hospitals. All migrants can access SMRU clinics and non-governmental organization (NGO)-run hospitals such as the Mae Tao Clinic (in Mae Sot) for free, but unstable migrants tend not to access these facilities due to travel and cost barriers.

The hardships the migrant community face are significant, but many women we spoke with also find ways to navigate these challenges by relying on support networks, including family, employers, community leaders, NGOs and research networks. Some of their employers provide basic housing, protection from authorities and assistance with health problems. Researchers in one FGD explained:

*0053: Some of the pregnant women, they'll tell you…I have a good boss, he has a car…sometimes the boss will take them on to the Thai hospital… (FGD, researcher)*

**Figure 2.**
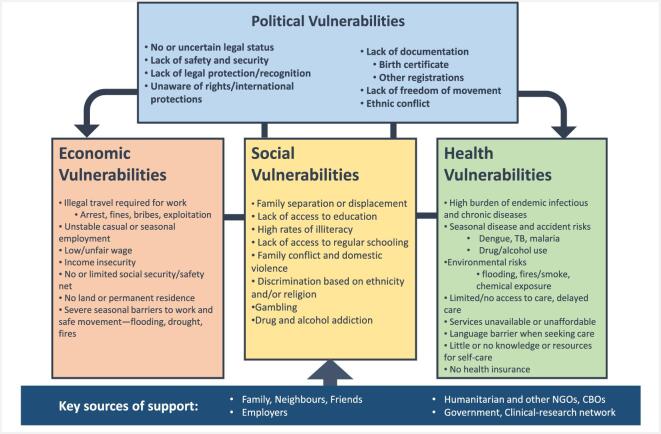
Intersectional structural vulnerabilities for migrants living along the Thai–Myanmar border.

Overall, migrant women participating in research, and their families, face political, economic and social vulnerabilities in their daily lives and these combine and contribute to critical health vulnerabilities. Despite the complex vulnerabilities, they relied on networks of social support, including family, employers and clinic and research staff. The relationship of cumulative sources of vulnerability and mitigating support are described in Figure 2.

### Agency, resourcefulness and perceived benefits of research participation

Migrant women's own accounts of assessing perceived benefits and deciding whether to participate in research demonstrated their agency and resourcefulness in navigating challenges in their lives. Against the backdrop of complex and chronic difficulties in daily life, many migrants at the Thai–Myanmar border go to great lengths to participate in research, making difficult journeys and attending follow-up visits. We asked migrant women why they joined research and what benefits they perceived in participation. Many migrants decide to participate in research because it is seen as being an important means of obtaining high-quality healthcare, a way to obtain extra money and a way to gain knowledge.

As described, many migrants have limited access to quality healthcare, much less affordable care. Employers, community leaders and others along the border region refer migrants to the SMRU clinics for care.

*008: When I got pregnant, my employer said, there is a clinic across the river, go there. I also have friends that got pregnant. They told me, you can go there, you can get checked there. (FGD, DMA and TDF participants)*

The SMRU is the only way to access free care for most migrants, and while they do not have to join studies to access these services, some participants viewed research as offering direct healthcare for their children. As this participant discussed:

*0010: It was good, they looked after us…I don't know how to care for my child at home…My child was small, and I don't know how to give them medicine…did not have to pay or anything. It's a big relief. (FGD, DMA participant)*

Antenatal care and deliveries are free at SMRU clinics, as is treatment for malaria. In the randomised trial of 3 artemisinin combination therapy for malaria in pregnancy (DMA) study, participants (pregnant women who have malaria) and their babies are followed up to 4 y, which is longer than the usual routine baby follow-up. Although they have to return to the clinic for regular study-specific check-ups, many said this gives them peace of mind and reassurance that their babies are healthy. Some research participants said the payment for participation is helpful. In some studies, the women receive goods as a token of appreciation, and they manage to exchange these for items at local shops. Even small amounts of money can go a long way for a family.

*0024: The [compensation] is helpful…I can buy flavouring powder, salt… (IDI, DMA participant)*

There are high levels of illiteracy among the migrants and they lack the opportunity for education. Some women we spoke to viewed research participation as a way to gain knowledge.

*0058: I like joining all kind of these groups…to get some kind of knowledge…for things that I never came across…for knowledge, I can learn about them. (IDI, TDF participant)*

When we asked women about decision-making in research, we found that all of them made their own decision to participate in research. None of the women interviewed said that they asked for their husband's or anyone else's permission.

*I: Did you decide on your own? Did you discuss with your husband?*

*0005: Decided on my own.*

*0011: I joined by myself.*

*0010: I joined by myself, my husband wouldn't know if you asked him.*

*(FGD, DMA participants)*

Migrant women further explained that most of the time, women make health decisions and decisions related to their children instead of their husbands.

While researchers sometimes questioned if consent for participation is truly voluntary, participants said they can say no. One participant told us that she declined to join a study due to the amount of medicine that needed to be taken but she gave another reason.

*0005: My son was almost one year old, and [name of researcher] called me [to clinic] and asked if I would join another study [not TDF or DMA]. I would have to take medicines and they want to know if I can tolerate the medicines or not. I looked at the bag of medicines [laugh], it was a lot [laugh]…I told a lie…that I will go back to Myanmar…[laugh]. (IDI, DMA participant)*

### The hidden burdens of research participation

While research at the border was perceived to be beneficial, it was also clear that research has the potential to exacerbate existing vulnerabilities. The dominant concerns reported by participants were the risks of travel across the border, foregoing casual labour, care for children left at home and being more visible as migrants.

*0012: I have to pick up my children, two of them. I need someone to help. I told [name of staff] that I cannot come…I have to pick up my children. She told me, please come, and I thought, not too bad, I can come but on one hand is my children. In the morning, I have to start cooking and at 7 a.m. I have to send them [to school]…pick them up at 12:30. There's no one else at home; my husband goes to the forest, goes to work and finds money…Money is short. (FGD, DMA participant)*

**Figure 3.**
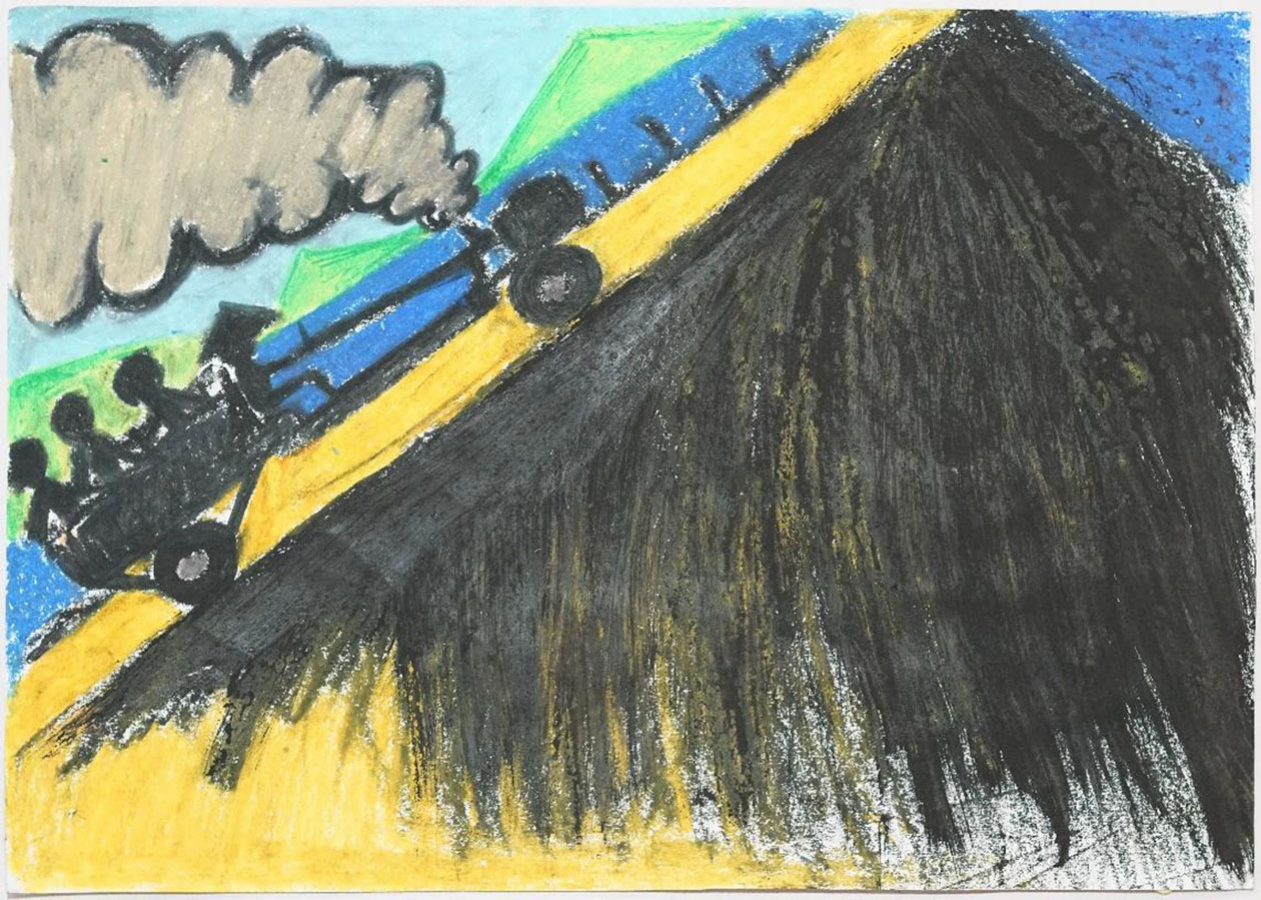
A drawing by the T-CAB members during a participatory visual workshop illustrating migrants’ difficult journey to the clinic.

Attending research clinic visits means that participants lose their wages for the day. Because travelling usually takes several hours, they may also struggle with childcare and other household duties. In addition, the more they have to travel, the more vulnerable they are to travel-related risks. A typical journey may include four or five modes of transport, including often piling on a farm tractor to get through muddy and hilly sections of road, as these T-CAB members illustrated (Figure 3).

### Ethical dilemmas arising for researchers

Researchers and frontline healthcare workers who implement research studies across the border clinic network described how this particular research network aimed to be directly responsive the health needs of the populations. As one healthcare worker explained, although the study does not offer direct individual benefit to the participant, the findings will have direct benefit to their community:

*0022: These medicines have been used but we want to know more on the efficacy for the pregnant woman…what is the dosage, for how many days, so they can be cured. It will also benefit other people who would come (to our clinic) in the future. (IDI, healthcare worker)*

Researchers working at the SMRU—a few of whom have lived and worked here for decades—shared their experiences of ongoing ethical dilemmas arising from the complex needs of migrants in the region and the perceived limitations of research to fully respond to these needs.

The first of the ethical challenges described related to balancing the benefits and burdens of research. For research to be ethical, most international guidelines mandate that the benefits of research must outweigh its burdens.^22^ But how much burden should an individual participant be allowed to take on for the future benefit of their own community? In the DMA study, women had to attend many follow-up visits after delivery to understand the effects of malaria and antimalarial drugs on child development when given during pregnancy. Although most participants expressed that they benefited from regular check-ups (e.g. peace of mind), they still had to make the difficult journey.

The second challenge is related to duty of care to research participants. The SMRU staff described themselves as part of the community with a strong sense of duty to respond to needs. The SMRU setting is unique, as it combines research and humanitarian care. The research aims to address critical migrant health vulnerabilities—for example, malaria research conducted with the border population has significantly improved the treatment of drug-resistant malaria on the Thai–Myanmar border.^23^^,^^24^ Yet researchers said they sometimes struggle to balance their roles as healthcare providers and researchers, describing the challenges arising from blurred lines between research and clinical services. Attempts to carefully explain to participants the difference between research and clinical care, including that receiving clinical services is not contingent on research participation, has resulted in complicated and long consent documents mandated by local and international research ethics guidelines.

The third challenge discussed surrounds compensation for research participation. In order to address some of the burdens of research, compensation is usually provided to participants for procedures that are specific for the study, e.g. extra visits. How much compensation to provide is itself an ethical dilemma: too much and researchers worry about unduly influencing participants, too little and they risk exploitation.^25^ Most researchers we interviewed preferred to provide more compensation if the study can afford it to ensure that participants are not out-of-pocket.

The fourth challenge involves voluntary consent. Researcher concerns about the voluntariness of participation were exacerbated by a familiar cultural tendency of *kreng-jai* (Thai) or *arr-nar* (Karen/ Burmese), understood as ‘the desire to be self-effacing, respectful, humble, and extremely considerate, as well as the wish to avoid embarrassing others or intruding or imposing on them’.^26^  *Kreng-jai/arr-nar* can also mean participants want to reciprocate by participating in research as a show of gratitude for perceived benefits to themselves or their community. Researchers worried it may be culturally difficult for participants to say no when approached for research participation. As noted above, this did not reflect what the women participating in research described as artful ways of polite refusal or avoidance.

## Discussion

Our findings provide evidence to support arguments by bioethical scholars that population-based definitions of vulnerability fail to capture the complexity of research participation in context^10^^–^^12^ and that paying careful attention to specific contextual and structural vulnerabilities is vital. Our evidence also supports arguments in favour of the importance of recognising and supporting the agency and resourcefulness of those who may be vulnerable in specific ways and situations.^12^

Our findings show that researchers should be aware of specific background vulnerabilities that may result in hidden burdens in research. For migrants on the Thai–Myanmar border, daily challenges for research participants revolve around historical political structures, limited freedom of movement, poverty, seasonal challenges and poor access to healthcare. These are cumulative and reinforcing (Figure 2). Some of these challenges are likely to be similar in other low-resource settings, but researchers should also be aware of vulnerabilities specific to their community.

Research that is responsive to the health of special populations such as migrants and pregnant women remains important. As our study confirms, research is critical for providing evidence-based care targeting their needs. For this reason, it is vital not to categorically exclude groups of people such as pregnant women from research and not to exacerbate their existing vulnerabilities or create new ones in the course of conducting research. As our case study illustrated, this can mean minimising research follow-up visits, helping with transport wherever possible and ensuring that compensation for time and loss of work are adequate. Researchers and ethics committees often worry about providing payments for research. In our study we found that it is important that participants are compensated adequately to cover often hidden burdens.

Migrant women we heard from all made the decision to participate in the study on their own, choosing to do so to access the benefits of research. They made decisions and took actions that in many ways reflected agency and resourcefulness. However, we do not wish to overemphasise agency here—not only because of the ongoing presence of structural constraints in participants’ lives, but also because participants did not represent their actions as incredible or unusual. Instead, participants exhibited what Payne^27^ has characterized in other marginalised populations as ‘everyday agency’—the making of choices and navigation of challenges as part of everyday life, as opposed to something extraordinary. Participants described how research can offer many benefits, some of which researchers may not fully appreciate. Researchers and ethics committees should respect the agency of potential participants by making research available to them with adequate compensation for difficult journeys, missed casual work and child care. That being said, it is important to recognise that the agency of migrant women in this context is highly constrained by the lack of adequate health services and lack of free movement.^28^ This should be carefully weighed when higher study risks are involved.

While our study offers critical empirical data to inform research that is responsive to specific vulnerabilities, it also had some limitations. We aimed to recruit women's husbands to understand how research participation impacted the family. However, the men were usually at work. As such, we were unable to enrol as many husbands as hoped. We had also planned to compare refugee experiences with migrant experiences in research, however, we were only able to interview three refugees, because at the time of recruitment the SMRU was in the process of pulling out from the local (Mae La) refugee camp to focus on serving the migrant population. For this reason we focused our analysis on migrants.

## Conclusions

Our study confirms that research is important to provide evidence-based humanitarian care as well as offer important benefits to individual participants, but it also has hidden burdens. We also found that migrant women exercised agency and resourcefulness in navigating challenges in their lives and research participation.
